# SpinSPJ: a novel NMR scripting system to implement artificial intelligence and advanced applications

**DOI:** 10.1186/s12859-021-04492-y

**Published:** 2021-12-07

**Authors:** Zao Liu, Zhiwei Chen, Kan Song

**Affiliations:** 1 State Key Laboratory of Magnetic Resonance and Atomic and Molecular Physics, Wuhan Center for Magnetic Resonance, Wuhan Institute of Physics and Mathematics, Innovation Academy for Precision Measurement Science and Technology, Chinese Academy of Sciences, Wuhan, 430071 People’s Republic of China; 2 Zhongke-Niujin MR Tech Co. Ltd, Wuhan, 430075 People’s Republic of China; 3 Department of Electronic Science, Fujian Provincial Key Laboratory of Plasma and Magnetic Resonance Research, Xiamen University, Xiamen, 361005 People’s Republic of China

**Keywords:** NMR, Software, Script, Java, CPython, Instrument control, Data processing, Artificial intelligence

## Abstract

**Background:**

Software for nuclear magnetic resonance (NMR) spectrometers offer general functionality of instrument control and data processing; these applications are often developed with non-scripting languages. NMR users need to flexibly integrate rapidly developing NMR applications with emerging technologies. Scripting systems offer open environments for NMR users to write custom programs. However, existing scripting systems have limited capabilities for both extending the functionality of NMR software’s non-script main program and using advanced native script libraries to support specialized application domains (e.g., biomacromolecules and metabolomics). Therefore, it is essential to design a novel scripting system to address both of these needs.

**Result:**

Here, a novel NMR scripting system named SpinSPJ is proposed. It works as a plug-in in the Java based NMR spectrometer software SpinStudioJ. In the scripting system, both Java based NMR methods and original CPython based libraries are supported. A module has been developed as a bridge to integrate the runtime environments of Java and CPython. The module works as an extension in the CPython environment and interacts with Java via the Java Native Interface. Leveraging this bridge, Java based instrument control and data processing methods of SpinStudioJ can be called with the CPython style. Compared with traditional scripting systems, SpinSPJ better supports both extending the non-script main program and implementing advanced NMR applications with a rich variety of script libraries. NMR researchers can easily call functions of instrument control and data processing as well as developing complex functionality (such as multivariate statistical analysis, deep learning, etc.) with CPython native libraries.

**Conclusion:**

SpinSPJ offers a user-friendly environment to implement custom functionality leveraging its powerful basic NMR and rich CPython libraries. NMR applications with emerging technologies can be easily integrated. The scripting system is free of charge and can be downloaded by visiting http://www.spinstudioj.net/spinspj.

**Supplementary Information:**

The online version contains supplementary material available at 10.1186/s12859-021-04492-y.

## Background

Since its discovery in the 1940s, nuclear magnetic resonance (NMR) has been adopted in many important fields including chemistry, biology, medicine, etc. NMR software for spectrometers is an important tool to implement applications in these fields. With the expansion of NMR applications, a variety of usage scenarios have to be supported by the software. For example, a developer expects to prototype a spectral reconstruction method [1]; an NMR facility manager needs to handle data management; and a user wants to perform the statistical analysis [2] of a completed NMR experiment. To support above usage scenarios and rapidly developing NMR applications, emerging technologies have to be quickly integrated in NMR software. For instance, deep learning [3] has been successfully applied in non-uniform sampling [1], spectrum denoising [4], chemical shift prediction [5–8], etc. Multivariate statistical analysis [2] plays an important role in metabolomics [9, 10] to reveal the relationships between metabolites and significant issues such as diseases and biological processes. However, NMR users have to wait for software vendors to integrate newly developed functionality and distribute the new versions. As an alternative solution for NMR users who expect to freely implement their own customized functionality in NMR software, scripting systems offer an open environment that allows users to write scripting programs. As a module in NMR software, the scripting system can both extend the existing functionality of NMR software’s non-script main program and perform native libraries of scripting languages. In the scripting system, existing non-script functions such as instrument control and data processing of main program can be called as a script style. As emerging technologies are expected to play an increasingly important role to solve complex problems for advanced NMR applications, it is essential to enhance scripting systems’ capabilities of implementing emerging technologies and advanced applications.

To enhance the capabilities, existing scripting systems have adopted a variety of solutions. In general, the solutions can be divided into two types. For the first type, the scripting system runs as an extension of the main program which is compiled with another computer language. Most commercial NMR software is of this type. For example, MAGICAL, is supported in VnmrJ [11]. This software is based on the “shell” scripting language which is native in UNIX like operating systems. The macros of MAGICAL support complex pulse sequence and custom commands. Jython [12], Tcl [13], and the AU program [14] are supported in TopSpin [15]. Jython and Tcl are standard scripting languages; the AU program is based on the C programming language and macros, and it needs to be compiled in GNU environment. Mnova [16] uses the native scripting language of the Qt library [17], named QtScript, to call powerful NMR algorithms of C++ based programs. ACD/Spectrus Processor [18] supports a collection of standard scripting languages (e.g., BasicScript, PascalScript, JavaScript, and C++ Script) to sequentially perform data processing and analysis. For the second type, the entire NMR software is developed using a scripting language. Examples in this category include MatNMR [19], jsNMR [20], rNMR [21], and nmrglue [22], which respectively use Matlab, JavaScript, R language [23], and CPython [24–26] as the scripting language for NMR data post-processing. This approach takes advantage of the powerful scientific computing libraries and chart display capabilities that are available.

However, the existing scripting systems may be difficult to use in the development of extended functionality for main programs and to support emerging technologies such as deep learning and multivariate statistics analysis. The main program of NMR software for spectrometers is often developed with a mainstream non-scripting language; these programs are typically compiled to binary code and directly executed by computer for higher execution efficiency. Scripting languages [27] are dynamically interpreted to machine instructions by corresponding interpreters in real time. Script programs can be freely modified and executed without recompiling. For existing scripting systems, the first type uses standard scripting languages and supports extending the functionality of main programs, but it has a more limited selection of advanced algorithms such as fast numerical computation and deep learning. The second type is better in advanced numerical computation, but it has critical disadvantages in execution efficiency [27] and implementing complex graphical user interfaces. These are important in the data acquisition and real-time curve display for instrument control applications. Existing NMR software of the second type focuses on data processing; they are not intended for the instrument control of spectrometers. Therefore, it is necessary to design a new scripting system, which has the capabilities to extend the functionality of the main programs (written in non-scripting languages) and rapidly implement emerging technologies by using advanced script libraries.

In this paper, a novel NMR scripting system named SpinSPJ (SpinStudioJ' Scripting system with Python and Java) is introduced. By integrating the CPython and Java programming languages, the system offers the benefits of both languages. CPython has flexible syntax features and has been widely adopted in various fields such as artificial intelligence, scientific computation, etc. It has a rich collection of libraries and a powerful ecosystem, which is significant for developing NMR advanced applications; The Java programming language [28] is cross platform and robust enough to implement multithreading, complex graphical user interfaces, etc. It is the development language for the main program of SpinStudioJ [29], which is an NMR software for spectrometers. The use of Java language is beneficial for the scripting system to interact with the main program. Therefore, SpinSPJ can extend the functionality of SpinStudioJ’s main program such as instrument control, data acquisition and data processing implemented in Java; in addition, it can rapidly adopt emerging technologies by leveraging CPython’s rich native libraries.

## Implementation

### Architecture

**Fig. 1:**
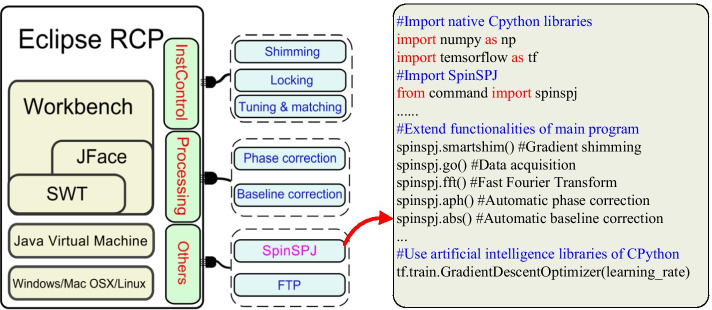
The relationship between SpinSPJ and SpinStudioJ. SpinStudioJ is a NMR software for spectrometers. It is built with a plugin architecture called Eclipse Rich Client Platform which integrates the functionalities as a plugin style. SpinSPJ works as a plugin in SpinStudioJ. SpinSPJ can call the Java based functions of instrument control and data processing, as well as using the native libraries of CPython

The proposed scripting system SpinSPJ offers a flexible scripting environment to implement custom functionality for NMR users. SpinSPJ works as a plug-in in SpinStudioJ, which is a plug-in based NMR software. SpinSPJ interacts with other plug-ins with the mechanism defined by framework OSGi (Open Service Gateway Initiative) [30]. The relationship between SpinSPJ and SpinStudioJ is illustrated in Fig. 1.

The conventional instrument control and data processing capabilities are implemented in Java. CPython has advantages in the availability of advanced libraries for numerical computation and artificial intelligence (e.g., NumPy [31], TensorFlow [32]). The significant issue of the proposed scripting system focuses on how to build a bridge that connects Java and CPython so that both Java-based NMR methods and third-party CPython libraries are supported. CPython, developed with the C programming language, supports an extension mechanism to wrap C libraries as customized modules. The Java virtual machine provides a mechanism called the Java Native Interface (JNI) [33] to support interactions with the C programming language. Through the JNI, Java can call functions defined by C, and C also can access resources (e.g., classes, functions, objects) in the Java environment. Therefore, the C programming language can serve as an ideal bridge between the Java and CPython languages.

**Fig. 2:**
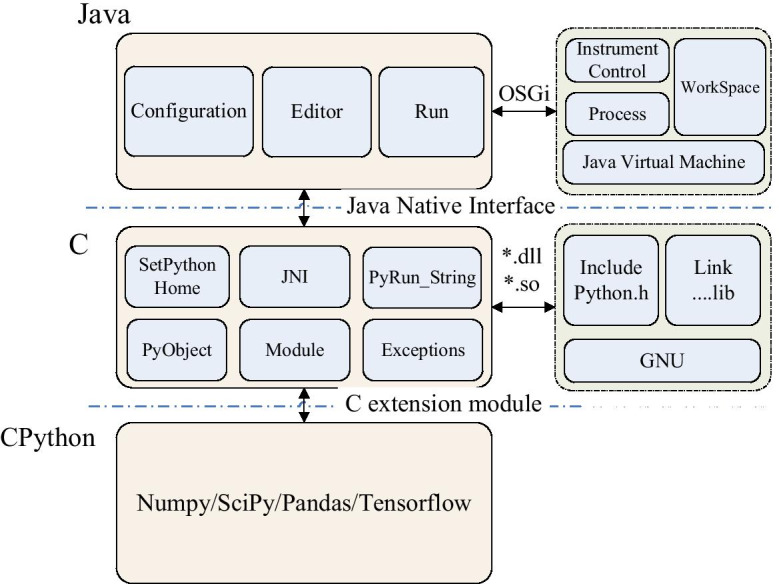
Architecture of the proposed SpinSPJ scripting system. The three components play different roles in the scripting system. Component Java extends the functionality of SpinStudioJ’s main program by the mechanism of OSGi. Component CPython offers the powerful libraries such as Numpy, SciPy, Pandas, Tensorflow, etc. Component C works as a bridge to connect Java and CPython to make their resources accessible to each other

The overall architecture of SpinSPJ is illustrated in Fig. 2. According to the computer languages adopted, the entire scripting system consists of three components: Java, C, and CPython.

The Java component is responsible for the graphical user interface and provides the interfaces for CPython including the basic configuration, scripting editor, instrument control, and data processing. The basic configuration can set the location of CPython libraries. The scripting editor offers a script editing window, execution output, menus, and toolbar. The interfaces for instrument control and data processing are implemented by the OSGi which separates the abstract interface from the concrete business logic. The instrument control includes sample control, temperature control, tuning, locking, shimming, data acquisition, etc. The data processing includes Fourier transform, phase correction, baseline correction, peak picking, and integration functionality. The NMR data of a HDF5 [29, 34] based custom format organises parameters, pulse sequence, free induction decay (FID), spectrum, peak list as a hierarchical style in a single file. It can be easily read and converted to other data formats by third party analysis tools (e.g., Matlab, CPython). After processing operations selected by NMR users are performed by CPython scripts, the processed data can be written back and saved to the disk. The scripts can be set to both blocking and non-blocking modes to ensure the statements are executed in the expected sequence.

The C component is the bridge between the Java and CPython components. Through the JNI, Java can call functions in C based libraries. If necessary, the C component also can create Java objects and call Java functions. Based on the C extension mechanism of CPython, the C component can define customized modules for the CPython component. In the CPython environment, customized modules can be compiled and linked with basic CPython libraries. Two significant methods used to accomplish these are the SetPythonHome method (set the location of the CPython libraries) and the PyRun_String (execute scripted code). A Java object can be wrapped as a PyObject, which is the foundation of the CPython language. As Java based resources can be accessed by CPython, the environments of Java and CPython are connected by the C component. In SpinSPJ, the C component creates a C extension module of CPython and builds the Java-CPython bridge with the help of an open source library called Jep [35].

The CPython component can define customized initialization and import methods for packages and modules, as well as offering various native libraries (e.g., NumPy, SciPy, TensorFlow). The initialization and import are the significant steps which enable the interactions between CPython and Java. For native libraries, NumPy and SciPy are usually used for fast numerical operations and scientific calculations. TensorFlow is widely used for deep learning. Non-uniform sampling and chemical shift prediction methods developed by deep learning can be easily integrated into the scripting system.

### Workflow

The workflow of SpinSPJ explains how the internal components work from the perspective of a time series. It contains the sequential actions and interactions of the components Java, C, and CPython during different stages. The workflow consists of three main stages: initialization, execution, and exiting.

The initialization stage is mainly for the preparation of the scripting environment. In this stage, the scripting system first configures the location of the CPython libraries. Secondly, CPython installs an importer hook and inserts it into the sys.meta_path. The importer hook defines the methods find_module and load_module to tell CPython how to find and load Java packages. Therefore, the resources of Java and CPython have been connected and the scripting environment has been established in this stage.

**Fig. 3:**
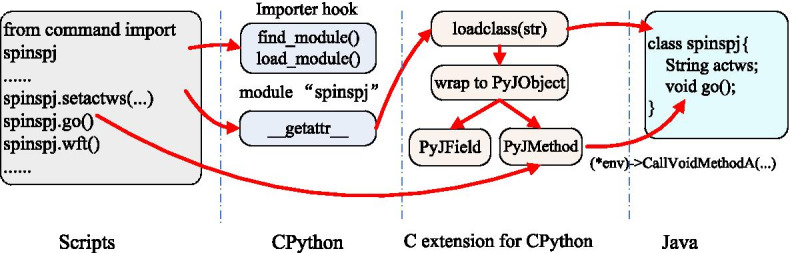
Execution workflow of the proposed SpinSPJ scripting system. The arrows denote the execution flow of script statements in each components. Firstly, by importing the module spinspj, the scripting system installs an importer hook, which defines how to find (“find_module()”) and load (“load_module()”) the module spinspj. Secondly, the fields and methods of module spinspj are wrapped as PyJField and PyJMethod, which enable the Java based fields and methods being called as a conventional CPython style through the JNI

In the execution stage, the scripting system controls the three components to support grammar features and resource accessibility. The workflow of the stage is illustrated in Fig. 3. There are two significant issues in this stage: import and interpretation. Different from conventional CPython environments, import statements in SpinSPJ can be used to import Java packages. When an import statement is called, the scripting system searches for the expected package from the variable sys.modules. If the package is found, it indicates that the package has been loaded by CPython; otherwise, the CPython environment finds the importer hook to invoke the find_module and load_module methods to load the spinspj module. The spinspj module is used to interact with Java resources. The spinspj module has a method __getattr__ to define its submodules for packages and classes in Java environment. The methods (wrapped to PyJMethod) and fields (wrapped to PyJField) of Java objects are wrapped as the attributes of a CPython object. CPython allows the PyJMethod to implement custom execution by defining the attribute tp_call of PyTypeObject, and allows PyJField to implement custom getting and setting styles by defining the attributes tp_getattro and tp_setattro of the PyTypeObject. An example is illustrated in Fig. 3. When the NMR command go is executed in scripts, PyJMethod invokes corresponding Java method by the JNI. Therefore, the CPython interpreter can recognize Java objects as conventional native CPython objects, as well as calling Java methods freely.

In the exiting stage, exception and memory management are the significant issues to ensure the scripting system is stable and robust. The JNI allows the C component to throw C based exceptions to the Java component. The Java component catches exceptions and back traces. For memory management, the Java component can reclaim memory at runtime by automatically leveraging the garbage collection feature of the Java virtual machine, so there is no need to release memory manually. However, the C component must release memory manually. Both the JNI and the C extension mechanism offer corresponding methods to release memory in order to avoid memory leaks.

## Results

**Fig. 4:**
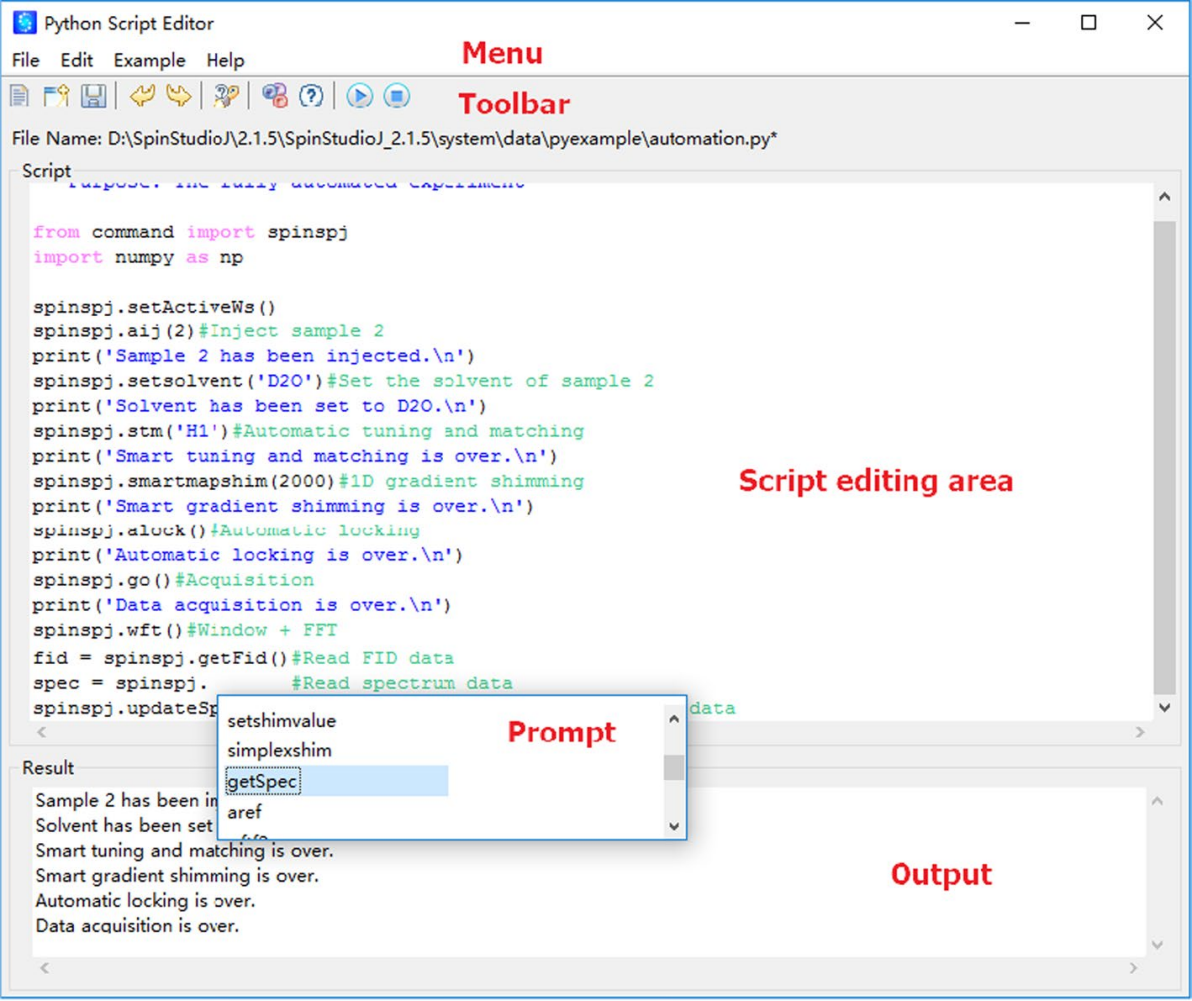
A screenshot of the NMR scripting editor. The scripting editor offers an environment to edit and execute the scripts of SpinSPJ. The script code and output are respectively displayed in the middle and bottom area of the editor’s interface

The proposed SpinSPJ scripting system provides a familiar graphical user interface layout for the user, which makes it straightforward to use. A screenshot of the NMR scripting editor is illustrated in Fig. 4. The menus and tools offer not only conventional functionality for file access and text editing, but also example scripts and help manuals. All the function and parameter definitions are described in the help manuals. In addition, commands for starting and stopping to run the scripts are available both in menus and toolbar. For script editing area, the key words of CPython can be marked as a highlighted style. Code comments and strings can be respectively displayed as green and blue. When NMR users enter the code spinspj., the available fields and methods of the spinspj module are displayed as prompts. When there is only one available option, the statement is automatically completed. The bottom region of the interface can show the outputs during the execution of scripts. The reported errors, warnings, and exceptions can prompt users to deal with problems during the execution of script programs. The script file (e.g., file name is “xxxx.py”) can be executed by entering the file name (e.g., “xxxx”) of the script in the command line of NMR software SpinStudioJ.

The scripts offer general functionality such as instrument control, data processing as well as native CPython libraries. Typical scripting functions are described as Table 1.

**Table 1:** General functions in the proposed SpinSPJ scripting system

Function name	Description	Example
*Instrument control*		
aij(n)	Inject a sample, The sample number is “n”	aij(2)
alock()	Lock the field automatically	alock()
stm(nucleus)	Automatic tuning and matching	stm('H1')
smartmapshim()	Create a gradient shim map and perform the shimming	smartmapshim()
smartshim()	Perform the gradient shimming using the existing field map	smartshim()
searchshim(algorithm, evaluation, channels, iteration)	Searching for better shim values with an algorithm	searchshim('simplex', 'FIDArea', 'z1-z2', 50)
vartemp(target, timeout)	Vary temperature to “target” celsius degree within “timeout” seconds	vartemp(35.5, 240)
spin(target, timeout)	Rotate the sample with the spin rate of “target” Hz within “timeout” seconds	spin(20, 200)
setshimvalue(channel, value)	Set the shim value of shim coil in “channel”	setshimvalue('z1', 1000)
go()	Start the data acquisition	go()
*Data processing*		
setactws(path)	Set the active workspace	setactws('D:/1.nmr')
setparam(name, value)	Set the value of parameter of the active workspace	setparam('ns', 4)
getfid(path)	Get the FID data of the workspace whose storage path is “path”	getfid('D:/1.nmr')
getspec(path)	Get the spectrum data of the workspace whose storage path is “path”	getspec('D:/1.nmr')
setspec(path, data)	Set the spectrum data of the workspace whose storage path is “path”	setspec('D:/1.nmr', data)
wft()	Perform the data processing with weighting and Fourier transform	wft()
*Original CPython libraries*		
np.multiply(a, b)	Matrix multiplication	np.multiply(a, b)
np.median(a)	Compute median of an array	np.median(a)
plt.plot(x, y)	Draw a curve	plt.plot(x, y)
scipy.optimize.curve_fit (func, x, y)	Compute curve fitting	scipy.optimize.curve_fit(func, x, y)

Instrument control is used to control the NMR spectrometer’s physical components such as auto sample changer, temperature control, shimming, and locking units. For data acquisition, scripts are allowed to set the parameters of the workspace, and then start the acquisition command. Both the blocking and non-blocking modes are supported. In the blocking mode, the script waits for the completion of the invoked method until the maximum time is exhausted. In the non-blocking mode, the script invokes the method and doesn’t wait for the completion of its execution.

For data processing, scripts can call conventional processing methods such as linear prediction, Fourier transform, phase correction, baseline correction, etc. Scripts can access an FID or spectrum from a workspace, as well as updating the spectrum display after a transformation or analysis.

In order to achieve powerful performance, most native libraries of CPython can be used in the scripting system, including NumPy, SciPy, matplotlib, and TensorFlow. NumPy supports a user-friendly and efficient numerical manipulation. Users can read data (FID or spectrum) from a workspace and convert the data format to an ndarray or matrix; these are the basic data formats for fast numerical calculations in Numpy. SciPy is a scientific computation library which can be used in parameter optimization and data denoising. Matplotlib library is a visualization library for FID or spectrum plotting. TensorFlow can be used to implement deep learning which is an emerging field in NMR.

**Fig. 5:**
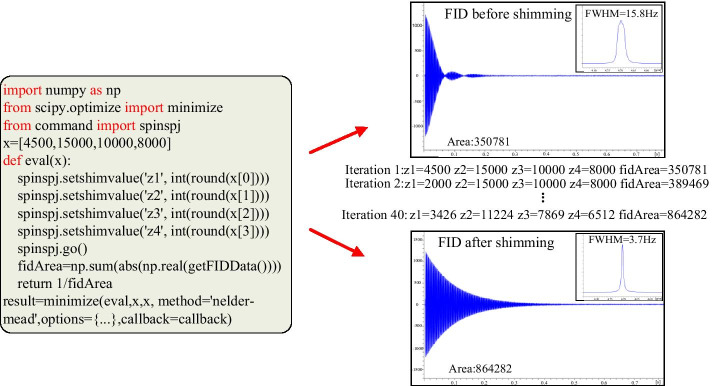
Searching shimming by FID area and simplex algorithm. As the evaluation of field’s homogeneity, FID area can be acquired and calculated by calling Java based method; the library SciPy of CPython provides the simplex algorithm to optimize the shim values with an iterative style. The homogeneity of the magnetic field is much better after utilizing the searching shimming method

To illustrate the performance of the scripting system, three examples of NMR methods are presented. The first script example for automatic searching for shimming is illustrated in Fig. 5. Automatic searching for shimming aims to calibrate output values of instrumental shim power supply by using a multivariate optimization algorithm, which can significantly improve the homogeneity of a center magnetic field. It needs real-time data acquisition and optimization analysis with an alternating style. The proposed SpinSPJ scripting system offers Java based instrument control for setting shim values and real-time data acquisition. The SciPy library provides the optimization algorithm Simplex [36] to generate new shim values with an optimized search path. As illustrated in Fig. 5, the experiment of searching for shimming has been performed on a Zhongke-Niujin 500 MHz QOne^Plus^ NMR spectrometer. The test sample is 0.1 mg/ml GdCl_3_ in D_2_O with 1% H_2_O. The evaluation criterion of field homogeneity is the area of FID, which can be affected by adjusting the shim values. In each iteration, Simplex can simultaneously change all of the Z1-Z4 shim values and seek their best combination by evaluating the area of the FID. After 40 iterations, the area of the FID is optimized from 350,781 to 864,282 (ADC value), and the half peak width of H_2_O peak is optimized from 15.8 to 3.7 Hz, which presents a substantial improvement on the magnetic field homogeneity. This example demonstrates that the scripting system can be used to flexibly develop user defined NMR methods for the instrument calibration by combining data acquisition and CPython based optimization algorithms.

**Fig. 6:**
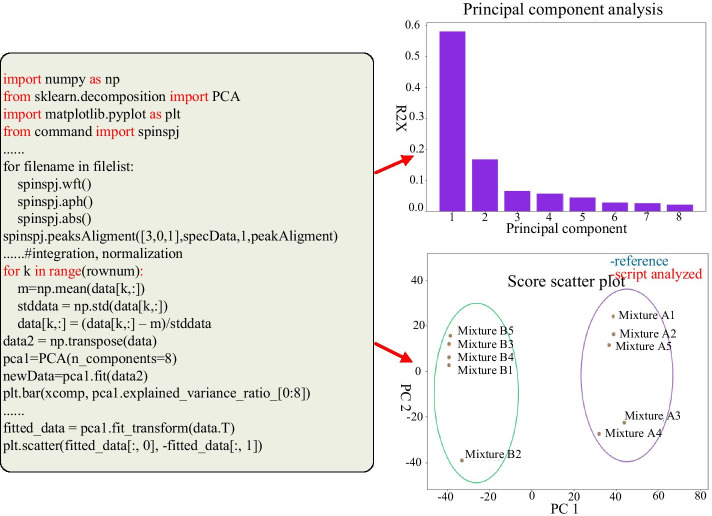
Principal component analysis. After some basic data processing, PCA is easily performed with the help of the CPython library scikit-learn

The second example is for a principal component analysis (PCA) [37] of metabolomic data. A PCA is the significant step to distinguish among the bulk data by projecting them onto multiple orthogonal components. As illustrated in Fig. 6, the data are from the web site metabolomics workbench [38], and the corresponding study ID is “ST000101” which presents an NMR analysis of synthetic mixtures. Each of a total of 10 samples has 20 synthetic metabolites. All of the samples can be divided into two groups because the quantities of the 10 metabolites are quite different. After a Java based automatic phase, baseline correction, and integration of binned spectrals, the data set is analyzed by a PCA that is implemented with the proposed SpinSPJ scripting system. The script for the PCA includes reading the original data, calculating the average and standard deviation, conducting a PCA, and displaying the columnar and scattered data. The CPython libraries of NumPy, scikit-learn[39], and matplotlib are helpful to implement above requirements. The histogram gives the result of the 1st–8th principal components and their contribution percentages. Among all of the components, the first principal component can explain 58.02% of the information in the total samples. The score scatter plot shows the score values of each sample on the first and second principal component, where “·” denotes the published result of the study and “×” denotes the calculated score values by the scripts. Obviously, on the first dimension of the PCA, the samples are divided into group A and B, which is consistent with the metabolite composition of the samples. The data of the score scatter plot are consistent with the published results of the study [40]. This example indicates that the scripting system can support complex multivariate statistical algorithms and chart displays with the help of native CPython libraries.

**Fig. 7:**
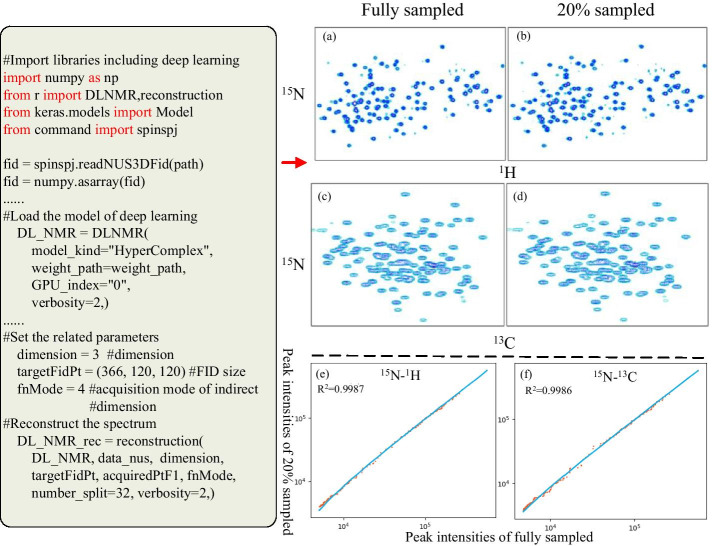
Non-uniform sampling by deep learning. The original FID data is read by calling the function offered by the main program of SpinStudioJ. The reconstruction method based on deep learning is easily performed with the help of CPython libraries such as Tensorflow, keras, cuDNN, etc.

The third example focuses on deep learning NMR (DLNMR) [1] for non-uniform sampling (NUS)[41, 42]. NUS is an emerging NMR field that can accelerate multi-dimensional experiments of biomacromolecule as well as reducing the heating effect due to RF excitation. Deep learning based NUS methods have achieved accurate and fast reconstruction. The script reconstructs the spectra with a smart dense convolution neural network (DCNN) [1], which has been trained with simulated or acquired data. A DCNN requires a variety of libraries to implement the convolution and data fitting capabilities. SpinSPJ can integrate all of the related libraries (such as TensorFlow, keras, cuDNN) in the CPython environment. As illustrated in Fig. 7, a 3D HNCO NMR data of Azurin (molecular weight is 14 kDa) with full sampling was downloaded from the MddNMR website http://mddnmr.spektrino.com. A fair comparison between full and 20% sampling with respect to the spectral quality are shown in (a)–(f). In Fig. 7, (a) and (c) are the sub-regions of the projections on the planes of ^15^N–^1^H and ^15^N–^13^C for the fully sampled 3D spectrum, which is reconstructed by fast Fourier Transform. (b) and (d) are the corresponding reconstruction results of a 20% sampling rate, which is reconstructed by a DLNMR method. (e) gives the correlation coefficient of the peak intensities of (a) and (b); (f) gives the correlation coefficient of the peak intensities of (c) and (d). The correlation coefficients of peak intensities between DLNMR reconstructed and fully sampled spectrums are greater than 0.99, which indicates excellent fidelity of the two spectrums. For the 3D NMR, the achieved acceleration factor of 5 in NUS implies that the experimental time can be reduced from 22.4 to 4.48 h. In addition, the computation time for the 3D reconstruction is 9.66 s (data size: 732*60*60, GPU: NVIDIA Tesla K40m), which demonstrates the method achieves very high computational efficiency. This example shows the capability of the scripting system to implement a deep learning based NMR method by leveraging native CPython libraries.

## Conclusion

SpinSPJ is a novel NMR scripting system which offers general functionality for instrument control and data processing; in addition, it can leverage native CPython libraries. Conventional instrument control and data processing are implemented by Java programming language; CPython libraries are helpful for providing advanced algorithms such as fast numerical computation, artificial intelligence etc. More advanced NMR functionality such as chemical shift and protein structure prediction are going to be integrated in the future.

SpinSPJ can be downloaded free of charge by visiting the website: http://www.spinstudioj.net/spinspj. The source code is private and owned by Zhongke-Niujin MR Tech Co.Ltd. The released software products are freely available to any researcher wishing to use them for non-commercial purposes, and licenses are needed for commercial purposes. The coded scripts are available in the GitHub repository https://github.com/qonenmr/spinspj.

### Availability and requirements

Project name: SpinSPJ

Project home page: http://www.spinstudioj.net/spinspj

Operating system(s): Platform independent

Programming language: Java, CPython

License: source code private

Any restrictions to use by non-academics: license needed

## Supplementary Information

**Additional file 1: User manuals**. Instructions about the installation, function list and programming for the proposed scripting system SpinSPJ.

**Additional file 2: Script examples**. Example script files of SpinSPJ, including automation, searching shimming, baseline correction, PCA, NUS, etc.

## Data Availability

All of the programs and script examples can be downloaded by visiting web site: http://www.spinstudioj.net/spinspj. The coded scripts are available in the GitHub repository https://github.com/qonenmr/spinspj. The original data for PCA are from https://www.metabolomicsworkbench.org/data/pca/show_metabolite_pca_NMR.php. The NMR example data of sample Azure for NUS have been downloaded from http://mddnmr.spektrino.com.

## Abbreviations

NMR: Nuclear magnetic resonance
NUS: Non-uniform sampling
MAGICAL: MAGnetics instrument control and analysis language
FID: Free induction decay
JNI: Java native interface
OSGi: Open Service Gateway Initiative
DCNN: Dense convolution neural network
PCA: Principal component analysis
DLNMR: Deep learning NMR
